# Expression of Extracellular Matrix-Related Genes and Their Regulatory microRNAs in Problematic Colorectal Polyps

**DOI:** 10.3390/cancers12123715

**Published:** 2020-12-11

**Authors:** Margareta Žlajpah, Emanuela Boštjančič, Bojan Tepeš, Nina Zidar

**Affiliations:** 1 Faculty of Medicine, Institute of Pathology, University of Ljubljana, 1000 Ljubljana, Slovenia; margareta.zlajpah@mf.uni-lj.si (M.Ž.); emanuela.bostjancic@mf.uni-lj.si (E.B.); 2 Gastroenterology Unit, AM DC Rogaška, 3250 Rogaška Slatina, Slovenia; abakus.medico.doo@gmail.com

**Keywords:** extracellular matrix, colorectal carcinoma, adenoma with epithelial misplacement, adenoma with early carcinoma

## Abstract

**Simple Summary:**

During bowel cancer screening programs, many diagnostically problematic polyps are removed. The greatest challenge is to distinguish between adenomas with epithelial misplacement and adenomas with early carcinoma, considering the diagnosis affects prognosis and treatment. Our aim was to analyze the expression of extracellular matrix related genes and proteins DCN, EPHA4, FN1, SPARC, SPON2, and SPP1, in 44 biopsies. Differences were observed in most of the analyzed genes and proteins in adenoma with epithelial misplacement in comparison to adenoma with early carcinoma, reflecting inflammatory stromal reaction to traumatisation and misplacement of dysplastic glands in the submucosa in the former, and desmoplastic stromal reaction to true invasion of dysplastic glands in the submucosa in the latter. The observed expression patterns are too complex to be used in diagnostic work, but might contribute to better understanding extracellular matrix changes in colorectal cancerogenesis and help to find new diagnostic markers in the future.

**Abstract:**

Colorectal carcinoma usually evolves gradually, forming a spectrum of lesions, due to accumulation of genetic mutations and epigenetic alterations. Many early lesions are detected since the introduction of screening programs. The greatest challenge is to distinguish between adenomas with epithelial misplacement (AEM) and adenomas with early carcinoma (AEC), considering the diagnosis affects prognosis and treatment. We analyzed the expression of selected extracellular matrix (ECM)-related genes and proteins, and their regulatory microRNAs using RT-qPCR and immunohistochemistry in biopsies from 44 patients. Differences were observed in AEM in comparison to AEC for *DCN*, *EPHA4*, *FN1*, *SPON2*, and *SPP1*, reflecting inflammatory stromal reaction to traumatisation and misplacement of dysplastic glands in the submucosa in the former, and desmoplastic stromal reaction to true invasion of dysplastic glands in the submucosa in the latter. Expression of regulatory microRNAs *hsa-miR-200c* and *hsa-miR-146a* significantly negatively correlated with the expression of their regulated genes, while significant difference between AEM and AEC was observed only for *hsa-miR-29c*. The described expression patterns are too complex to be used in diagnostic work, but might contribute to better understanding ECM changes in colorectal carcinoma development, helping to find new markers in the future.

## 1. Introduction

Colorectal carcinoma (CRC) is a heterogeneous disease, which usually evolves gradually, forming a spectrum of lesions, due to accumulation of genetic mutations and epigenetic alterations in key growth regulatory and differentiation genes [1,2,3,4]. The correct histopathologic diagnosis of different stages of CRC is of vital importance enabling ¸to choose the optimal treatment. Endoscopic removal is the treatment of choice for adenomas and adenomas with epithelial misplacement (AEM), also referred to as pseudoinvasion, since these lesions do not metastasize and additional surgical treatment is not necessary. In contrast, adenomas with early carcinoma (AEC) are capable of metastasizing and in some patients, surgical removal of the affected bowel with regional lymph nodes is needed.

CRC screening programs worldwide have enabled to detect and remove a large number of early polypoid lesions, including adenomas, AEM and AEC [5]. In the majority of cases, histopathology examination is straightforward, but there is a growing number of cases with ambiguous histopathologic features. For this reason, we would need additional histopathologic, immunohistochemical and/or genetic markers to be used in problematic lesions [6,7]. The most challenging task is to distinguish between AEM and AEC. In both lesions, dysplastic glands are found in the submucosa, but only in AEC, it is the result of true invasion [7,8]. In AEM, dysplastic glands are present in the submucosa due to traumatization and consequent reparation. This is typically a result of intraluminal traumatic injury of the larger polyps due to combination of different factors (narrow, highly motile sigmoid colon, solid fecal material and diverticulosis) [6]. Histologically, true invasion is characterized by severe dysplasia and desmoplastic stromal reaction while in epithelial misplacement, dysplastic glands in the submucosa appear similar to the surface of adenoma and are usually accompanied by lamina propria [7]. Its characteristic features are also hemosiderin depositions and mucus lakes [7].

Markers to distinguish between AEM and AEC are lacking. Recently, microRNAs have been associated with the development of cancer, as they influence the expression of their regulated gene(s) [9]. In CRC, it has been shown that microRNA expression profile differs among the adenoma-carcinoma sequence [10,11]. We hypothesized that in terms of gene expression, AEM is similar to adenoma, and AEC is similar to CRC. In our previous study [5], we used a bioinformatics approach to identify candidate genes for biomarkers that would distinguish between adenoma and CRC. In this study, we analyzed the expression of extracellular matrix (ECM) related genes decorin (*DCN*), erythropoietin-producing hepatoma receptor A4 (*EPHA4*), fibronectin 1 (*FN1*), secreted protein acidic and cysteine rich (*SPARC*), spondin 2 (*SPON2*) and secreted phosphoprotein 1 (*SPP1*) in AEM in comparison to AEC. Moreover, using online databases, we predicted regulatory microRNAs for the aforementioned genes. We hypothesized that the expression of these microRNAs is simultaneously inversely proportional to the expression of mRNAs in the early stages of colorectal carcinogenesis.

## 2. Results

### 2.1. Patient Characteristics

Our study included biopsy samples from 44 patients and healthy colon mucosa samples from surgical margins from 21 patients. The gender and age of the patients are listed in Table 1. Fisher test for heterogeneity showed samples were heterogeneous when gender of the patients was compared among all groups. However, excluding adenoma group, samples were homogenous (*p* = 0.417). In the adenoma group, there were 4 tubular adenomas with high grade dysplasia, 4 tubulovillous adenomas with high grade dysplasia and 2 tubulovillous adenomas with low grade dysplasia. In the group of AEM, there were 1 tubulovillous adenoma with high grade dysplasia, 2 tubulovillous adenoma with low grade dysplasia, 3 tubular adenomas with high grade dysplasia and 7 tubular adenomas with low grade dysplasia. The AEC group consisted of 5 tubulovillous adenomas, 3 tubular adenomas, 1 villous adenoma and 1 tubular adenoma, all with high grade dysplasia and with malignant transformation, shown by invasion of the dysplastic glands in the submucosa (pT1). The pTNM stages in the last group were pT3N1Mx, pT4aN1Mx and pT4aN2Mx in 6, 2 and 3 patients, respectively.

### 2.2. Expression of ECM-Related Genes in Adenoma with Epithelial Misplacement Compared to Other Groups

**Figure 1:**
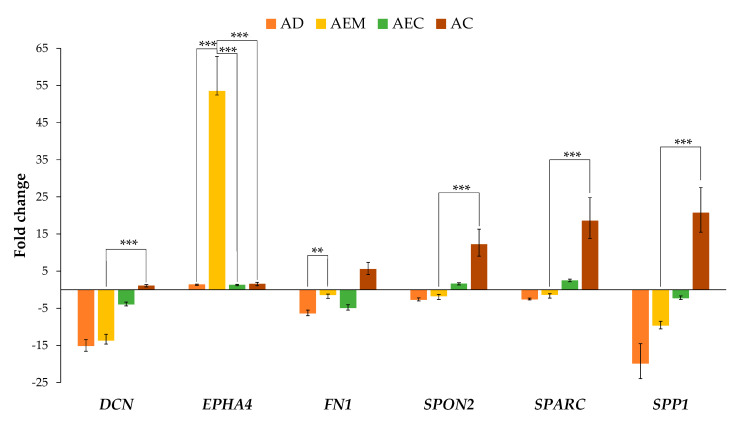
Expression of *DCN*, *EPHA4*, *FN1*, *SPON2*, *SPARC* and *SPP1* in adenoma, adenoma with epithelial misplacement, adenoma with early carcinoma and advanced carcinoma. Expression is plotted as fold change against healthy colon mucosa. Legend: *** *p* ≤ 0.001, ** *p* < 0.01; AD, adenoma; AEM, adenoma with epithelial misplacement; AEC, adenoma with early carcinoma; AC, advanced carcinoma.

The expression of the analyzed ECM-related genes, presented in Figure 1, showed down-regulation in adenoma in all genes except in the case of *EPHA4*, which was slightly up-regulated. Expression of *EPHA4* was also up-regulated in AEM, while expression of other genes in AEM showed down-regulation to a lesser extent than in adenoma. The difference in expression between adenoma and AEM was only significant for *EPHA4* (*p* < 0.001) and *FN1* (*p* = 0.009).

The expression pattern of the analyzed ECM-related genes showed that *DCN*, *SPARC*, *SPON2* and *SPP1* increased gradually with the level of malignancy as shown in Figure 1.

Among them, *SPARC* and *SPON2* were down-regulated in adenoma and AEM, and up-regulated in AEC and advanced carcinoma. Comparison of the expression level in AEM with the expression level in advanced carcinoma showed that the difference in expression was statistically significant for *SPARC* and *SPON2* (*p* < 0.001 for *SPARC* and *p* = 0.001 for *SPON2*).

The other two genes, *DCN* and *SPP1*, were down-regulated in adenoma, AEM and AEC, and up-regulated in advanced carcinoma. Statistical significance was found when comparing expression of *DCN* and *SPP1* in AEM and advanced carcinoma (*p* < 0.001 in both cases).

*EPHA4* showed similar expression in AEC and advanced carcinoma, while *FN1* showed down-regulation of expression in AEC and up-regulation in advanced carcinoma. In the case of *EPHA4*, comparison of expression of AEM to AEC showed that the difference in expression was statistically significant (*p* < 0.001). The comparison of AEM to advanced carcinoma was significant in case of *EPHA4* as well (*p* < 0.001).

### 2.3. Immunohistochemistry

**Figure 2:**
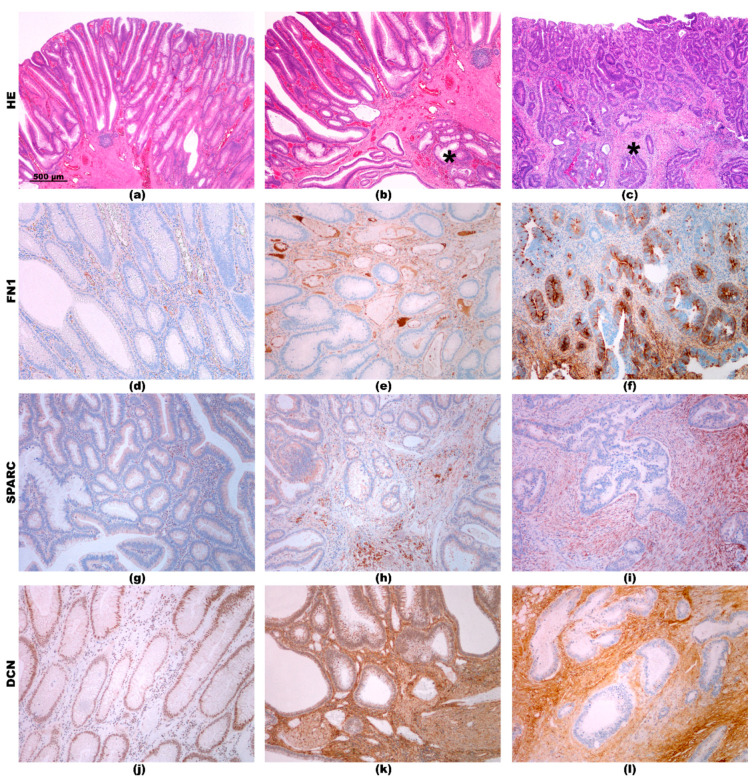
(**a**) Adenoma; (**b**) adenoma with epithelial misplacement—epithelial misplacement in the submucosa is marked by asterix *; (**c**) adenoma with early carcinoma; invasive carcinoma in the submucosa is marked by asterix *. HE staining, orig. magnification 4×. FN 1: Mild positive reaction in the stroma, mostly in capillaries, in adenoma (**d**). More intensive reaction in the stroma in epithelial misplacement (**e**). Focal reaction in carcinoma cells and in the stroma of invasive carcinoma (**f**). SPARC: Very mild positive reaction in epithelial cells in adenoma (**g**). Mild positive reaction in epithelial cells and focal positive reaction in the stroma in epithelial misplacement (**h**). Mild positive reaction in carcinoma cells and strong positive reaction in the stroma of invasive carcinoma (**i**). DCN: Mild positive reaction in epithelial cells in adenoma (**j**). Mild positive reaction in epithelial cells and strong positive reaction in the stroma in epithelial misplacement (**k**). No positive reaction in carcinoma cells and strong positive reaction in the stroma of invasive carcinoma (**l**). Immunohistochemistry, orig. magnification 10×.

**Figure 3:**
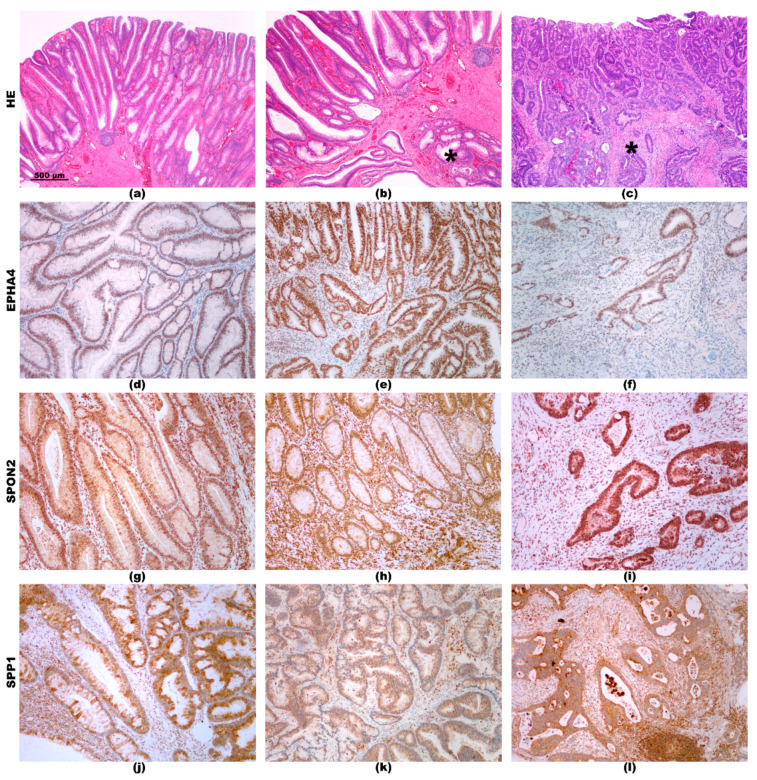
(**a**) Adenoma; (**b**) adenoma with epithelial misplacement—epithelial misplacement in the submucosa is marked by asterix *; (**c**) adenoma with early carcinoma; invasive carcinoma in the submucosa is marked by asterix *. HE staining, orig. magnification 4×. EPHA4: Mild positive reaction in epithelial cells in adenoma (**d**). More intensive reaction in epithelial cells and in the stroma in epithelial misplacement (**e**). Focal reaction in carcinoma cells and in the stroma of invasive carcinoma (**f**). SPON2: Positive reaction in epithelial cells and in the stroma in adenoma (**g**). Positive reaction in epithelial cells and in the stroma in epithelial misplacement (**h**). Strong reaction in carcinoma cells and positive reaction in the stroma of invasive carcinoma (**i**). SPP1: Positive reaction in epithelial cells and mild reaction in the stroma in adenoma (**j**). Positive reaction in epithelial cells and in the stroma in epithelial misplacement (**k**). Strong reaction in carcinoma cells and in the stroma of invasive carcinoma (**l**). Immunohistochemistry, orig. magnification 10×.

Immunohistochemical analysis of all 6 ECM-related proteins was performed for adenoma, AEM, and AEC, while for advanced carcinoma, it was described in our previous study [2]. Representative staining patterns for each protein are presented in Figure 2 and Figure 3.

Immunohistochemical reaction for FN1 in adenoma was very focal and faint, present in rare stromal cells and capillaries (Figure 2d). A more intensive reaction in stromal cells and capillaries was found in AEM (Figure 2e). In AEC, reaction in the stroma was even more intensive; additionally, focal positive reaction was also found in carcinoma cells (Figure 2f).

Immunohistochemistry for SPARC showed similar mild positive reaction in epithelial cells in adenoma, AEM and AEC. In the stroma, positive reaction was minimal in adenoma (Figure 2g), more intensive but focal in AEM (Figure 2h) and intensive, almost diffuse in AEC (Figure 2i).

Immunohistochemistry of DCN showed mild positive reaction in epithelial cells and in the stroma in adenoma (Figure 2j). In AEM, strong reaction was present in the stroma and mild in epithelial cells (Figure 2k). The same staining pattern for stromal cells was observed in AEC, whereas no positive reaction was present in carcinoma cells (Figure 2l).

Immunohistochemistry for EPHA4 showed positive reaction in epithelial cell in both adenoma and AEM, but the reaction in the stroma was mild in adenoma (Figure 3d), and more intensive in AEM (Figure 3e). In AEC, there was a partial loss of expression in carcinoma cells (Figure 3f).

Immunohistochemistry for SPON2 showed similar pattern in adenoma and AEM, being positive in both epithelial cell and in the stroma (Figure 3g,h). In AEC, reaction in carcinoma cells was more intensive than in adenoma and AEM (Figure 3i).

Immunohistochemical reaction for SPP1 was also similar in adenoma and AEM, in both epithelial cells and in the stroma (Figure 3j,k). In AEC, it was slightly more intensive both in carcinoma cells and in the stroma (Figure 3l).

### 2.4. Determining Regulatory microRNAs for the ECM-Related Genes

Potential regulatory microRNAs for the selected genes were chosen using different available online platforms and the available literature. We chose 11 different microRNAs and tested whether their expression was inversely proportional to the expression level of the regulated gene. Potential regulatory microRNAs for *SPP1* were determined in our previous study [12]. The chosen potential regulatory microRNAs for other ECM-related genes are listed in Table 2.

### 2.5. Expression of the Potential Regulatory microRNAs for the ECM-Related Genes

**Figure 4:**
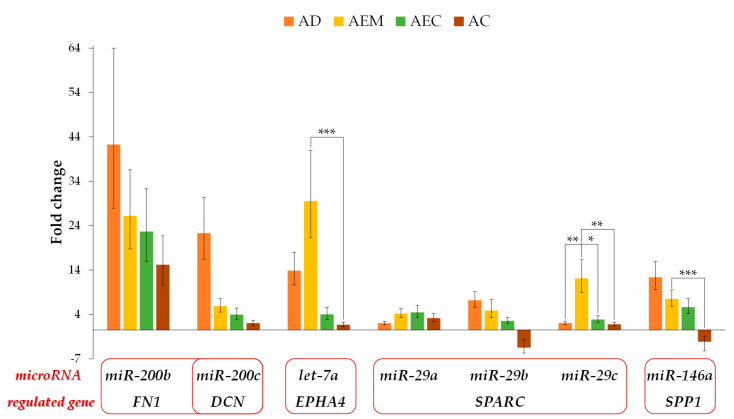
Expression of potential regulatory microRNAs in adenoma, adenoma with epithelial misplacement, adenoma with early carcinoma and advanced carcinoma. Expression is plotted as fold change against healthy colon mucosa. Legend: * *p* < 0.0125, ** *p* < 0.01, *** *p* ≤ 0.001; AD, adenoma; AEM, adenoma with epithelial misplacement; AEC, adenoma with early carcinoma; AC, advanced carcinoma.

Expression of the analyzed microRNAs is presented in Figure 4 and Figure S1. Expression of microRNAs in all 4 groups of the adenoma-carcinoma sequence was present in 7 out of 11 microRNAs that were analyzed. Expression of *hsa-miR-211* was present in healthy mucosa, adenoma, AEC, and advanced carcinoma but not in AEM. *hsa-miR-493* was only expressed in healthy mucosa and AEM. *hsa-miR-127* was expressed in AEC and *hsa-miR-299* was expressed only in adenomas and advanced carcinoma. Analysis of potential regulatory microRNAs showed that *hsa-miR-146a*, *hsa-miR-29a*, *hsa-miR-29b*, *hsa-miR-29c*, *hsa-miR-200b, hsa-miR-200c*, and *hsa-let-7a* were up-regulated in adenoma, AEM, and AEC. In advanced carcinoma, *hsa-miR-146* and *hsa-miR-29b* were down-regulated while other microRNAs were up-regulated. Significant difference between adenoma and AEM was observed only for *hsa-miR-29c* (*p* = 0.002). Expression of *hsa-miR-29c* between AEM and AEC was also statistically significant (*p* = 0.012). Comparing expression in AEM and advanced carcinoma, *hsa-miR-29c, hsa-miR-146a*, and *hsa-let-7a* were statistically significant (*p* = 0.002, *p* < 0.001, and *p* = 0.001, respectively).

### 2.6. Correlation between the Expression of Potential Regulating microRNA and Their Target ECM-Related Gene

**Figure 5:**
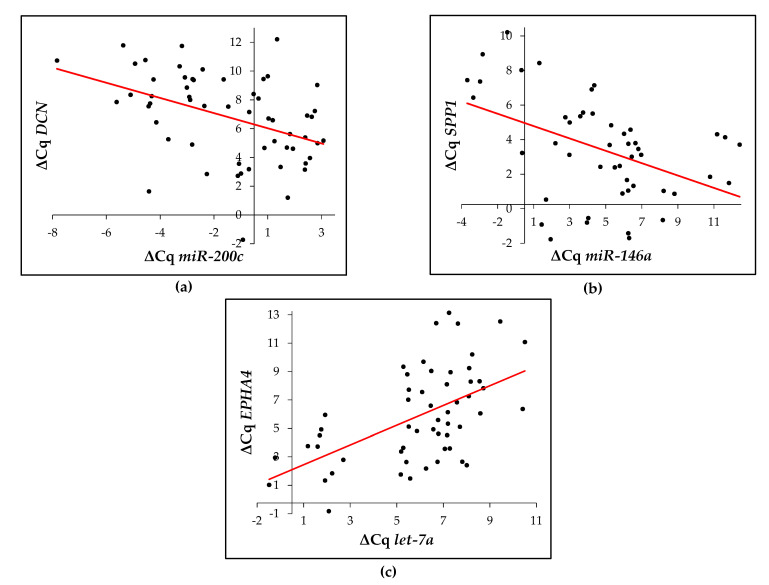
Representative figure of Spearman correlation coefficient for (**a**) *DCN*, (**b**) *SPP1* and (**c**) *EPHA4*.

We also checked if there is any correlation between the ECM-related genes and their potential regulatory microRNA. Statistically significant correlation was calculated between *hsa-miR-146a-5p* and *SPP1* (ρ = −0.607, *p* < 0.001) as shown in Figure 5a. Significant correlation was also observed for *hsa-miR-200c* and *DCN* (ρ = −0.509, *p* < 0.001) as shown in Figure 5b. Correlation between the expression *SPARC* and their potentially regulatory microRNAs was negative in case of *hsa-miR-29b* and *hsa-miR-29c*, and positive in case of *hsa-miR-29a* but not statistically significant. However, significant positive correlation was calculated for association between *EPHA4* and *hsa-let-7a* (ρ = 0.534, *p* < 0.001) as shown in Figure 5c.

## 3. Discussion

The aim of this study was to determine whether the expression of selected ECM-related genes (*DCN*, *EPHA4*, *FN1*, *SPARC*, *SPON2*, and *SPP1*) was similar in adenoma and AEM but differed from the expression in AEC and advanced carcinoma [2]. We found two genes, *DCN* and *SPP1*, showing different expression in AEM compared to either AEC or advanced carcinoma. Moreover, the expression of their regulatory microRNAs was significantly negatively (*hsa-miR-200c* for *DCN* and *hsa-miR-146a* for *SPP1*) or positively (*hsa-let-7a* for *EPHA4*) associated with the expression of their regulated gene.

Moreover, *SPON2* and *SPARC* showed up-regulation in AEC and advanced carcinoma, and down-regulation in adenoma and AEM. This expression pattern confirms our hypothesis that the expression of selected ECM-related genes is similar in adenoma and AEM, but differs from the expression in AEC and advanced carcinoma. Although the expression of one of the regulatory microRNAs for *SPARC* was inversely proportional to the expression of *SPARC*, statistical analysis did not show any significant correlation. Unfortunately, the expression pattern of other selected regulatory microRNAs for *SPARC* and *SPON2* was neither inverse nor statistically significantly correlated with their regulated gene.

Surprisingly, expression of gene *EPHA4* was highly up-regulated in AEM, where dysplastic glands are present in the submucosa due to traumatization and consequent reparation, in comparison to other lesions. Although its expression pattern did not confirm our hypothesis, its high expression indicates that *EPHA4* might serve as one of the markers of epithelial misplacement. So far, expression of *EPHA4* and of some other receptors of EPH family has been used do differentiate between various stages of non-small cell lung carcinoma. Additionally, their high expression correlated with low stage and presence of inflammation [30,31].

The remaining gene, *FN1*, did not show any pattern that would either confirm or reject our hypothesis. However, on the protein level, FN1 showed discrete differences between AEM and AEC in comparison to adenoma. Fibronectin promotes fibroblast migration directly, and promotes proliferation by regulating the bioavailability of TGFβ. Fibronectin also binds to TNFα, which promotes chemotaxis and expression of matrix metalloproteinase 9 in monocytes [32]. TNF in turn has an effect on the up-regulation of SPP1 [33,34].

ECM is formed by a diverse spectrum of molecules including proteins, proteoglycans, glycoproteins, and polysaccharides [35,36]. They provide unique biochemical, biophysical and biomechanical properties [37]. Under pathological conditions, the dynamics of the ECM changes. The main contributors of ECM remodeling are matrix metalloproteinases [35]. Study by Li et al. showed that expression of matrix metalloproteinases 2 and 9 increased in CRC compared to healthy colon mucosa [38]. Combination of functional domains, characteristic for matrix metalloproteinases, allows affecting several cellular processes, such as proliferation and apoptosis. Different experiments showed that degradation of surrounding tissue by matrix metalloproteinases have several functions that favor tumor progression by modulation of growth factors, inflammatory proteins, membrane receptors, adhesion molecules, and chemoattractants [39].

Inflammation is one of the pathological conditions that cause aberrant expression of ECM components. In inflamed tissues, cytokines e.g., TGFβ, TNF, and IFNγ cause protease secretion and initiate a cycle of ECM degradation and synthesis [33]. Consequently, proteases generate different chemotactic fragments that in turn recruit different immune cells to the site of the inflammation [32]. Collagen degradation products act as chemoattractant for neutrophil recruitment [35]. Therefore, the remodeled ECM of inflamed tissues affects the propagation of the inflammatory response and the development of the chronicity [33]. Tissues that are subject to chronic inflammation generally exhibit high cancer incidence [40].

In cancer, ECM becomes more stiff and rigid, which is among other the consequence of aberrant collagen and fibronectin deposition as well as excessive crosslinking by lysyl oxidases, as a result of desmoplasia [41]. Changes in the ECM stiffness causes the surrounding tissue to exhibit different biomechanical and biophysical properties, which in turn have, for example an effect on TGFβ signaling. Moreover, increase in collagen deposition up-regulates integrin signaling and can thus promote survival and proliferation. Therefore, the emerging environmental signals stimulate proliferative and apoptotic mechanisms, which are thought to lead to the selection of apoptosis-resistant cells with enhanced invasive potential [42]. The main contributors of altered activities of the ECM remodeling enzymes and ECM metabolism are stromal cells, including cancer-associated fibroblasts and immune cells. In advanced stages of cancer development, other cell types may also contribute to the altered composition of the ECM [36]. TGFβ is one of the essential cytokines that activate the fibrotic response and cancer stroma. TGFβ promotes myofibroblast differentiation and the recruitment of immune cells, inhibiting the anti-tumor immune responses and affecting epithelial and endothelial cell differentiation by controlling several different functions in most of the cells that form fibrous tissue [43].

The bioavailability and the downstream effects of TGFβ are lessened by binding of DCN to TGFβ reducing fibrous tissue [44,45]. Moreover, DCN might be one of the regulators of the synthesis of the ECM components and expression of collagenase, inhibitor of collagen I maturation which contributes to angiogenesis in the tumor [45]. TGFβ was also shown to stimulate SPARC function as an essential factor in tumor cell migration [46] where it participates as one of the regulators of the fibronectin network assembly. Otherwise, SPARC also participates to angiogenesis and wound healing [47]. A role in the immune response was also reported for SPON2 and SPP1. SPON2 participates in activation of immune response and recruitment of inflammatory cells [48], whereas SPP1 along with other pro-inflammatory factors contributes to tumor growth [49], angiogenesis by stimulating VEGF and macrophage recruitment [50]. SPP1 may be also involved in malignant transformation by transactivating different transcription factors. Moreover, SPP1 is one of the proteins needed for the process of fibroblast to myofibroblast differentiation [51].

Cell adhesion molecules on the endothelial cell surface interact with components of the ECM, such as fibronectin, collagens, and laminin to regulate both the recruitment of circulating leukocytes and modulate intracellular signaling pathways, which control endothelial permeability [32]. The EPH receptors are tyrosine kinase cell surface receptors that bind to their membrane bound ligands, ephrins, and modulate vascular permeability during inflammation [52]. It has been shown that up-regulated expression of *EPHA4* contributes to the spinal cord scar formation, since spinal cord injury in mice lacking expression of *EPHA4* resulted in axonal regeneration [52,53]. According to Ivanov et al., *EPHA4* receptor is mainly expressed in lymphocytes, monocytes, granulocytes and dendritic cells [54] and participates in regulation of T-cell development [52] and mediates T-cell chemotaxis [55]. Namely mice with *EPHA4* knockdown exhibited a blockage in T-cell maturation [52].

Our results indicate that some ECM-related genes could be post-transcriptionally regulated since they showed inverse correlation with their regulatory microRNA, e.g., *SPP1* and hsa-*miR-146a* [12] and *DCN* and *hsa-miR-200c*. Additionally, some microRNAs might be useful for distinguishing AEM from AEC, e.g., *hsa-miR-29c*. As with our bioinformatics approach, comparing adenoma to carcinoma to identify candidate genes to be used as markers for true invasion, bioinformatics analysis for identification of microRNAs to be used as markers would be also interesting. This approach was recently used by different research groups [9,10,11]. The true markers should be independent of specimens used [9,10]. Furthermore, inverse correlation between identified microRNAs and mRNAs would give us deeper understanding of the mechanisms of true invasion and epithelial misplacement.

The most important limitations of our study are the lack of functional validation of the analyzed microRNAs and a relatively small number of patients. The latter is due to the fact that our study included formalin-fixed paraffin-embedded (FFPE) tissue samples. In FFPE tissue, nucleic acids are fragmented and therefore difficult to analyze, but a great advantage of FFPE tissue is that samples are first evaluated by pathologists, enabling appropriate diagnosis. In our study, only samples that had successfully passed initial quality control and samples with stable expression of the reference genes were selected for further analysis, thus limiting the number of included samples. Furthermore, our study focused on problematic polyps, i.e., those containing either epithelial misplacement or early cancer, which usually occupy a small area, enabling a limited amount of appropriate tissue for analyses. For all these reasons, our results must be interpreted with caution.

## 4. Materials and Methods

### 4.1. Patients

The study was conducted in accordance with the Declaration of Helsinki, and the protocol was approved by the Ethics Committee of the National Medical Ethics Committee of the Republic of Slovenia (approval No. 0120-88/2018/4).

The study included biopsy samples from 44 patients divided into four groups based on the biopsy diagnosis: adenoma, AEM, AEC and advanced carcinoma. As a control group, healthy colon mucosa from surgical margins was included as well.

All tissue samples were fixed for 24 h in 10% buffered formalin and embedded in paraffin. For routine histopathological examination, 4 µm-thick slides were cut and stained with eosin and haematoxylin. For the present retrospective study, representative paraffin blocks were collected from the archives of the Institute of Pathology, Faculty of Medicine, University of Ljubljana.

### 4.2. Identification of the Potential Regulatory microRNAs

Different online platforms (TarBase, TargetScan, miRDB, miR-Tar and microRNA-mRNA expression atlas) were used to find microRNAs that could potentially regulate *DCN*, *EPHA4*, *FN1*, *SPARC*, *SPON2* and *SPP1* expression. We have considered only those interactions that were validated experimentally by direct methods such as reporter assay, western blot, or qPCR.

### 4.3. Isolation of RNA from Formalin-Fixed Paraffin-Embedded Tissue

For the extraction of total RNA, 10 µm-thick formalin-fixed paraffin-embedded tissue slides were used and RNA was extracted using AllPrep DNA/RNA FFPE kit (Qiagen GmbH, Hilden, Germany) following manufacturer’s protocol.

RNA concentration and quality was evaluated spectrophotometrically on NanoDrop^TM^ 1000 spectrophotometer (Thermo Fisher Scientific, Inc., Waltham, MA, USA). In all cases the A260/A230 intensity ratio was >1.0 and the A260/A280 > 1.8.

### 4.4. Reverse Transcription and Quantitative PCR

To measure the expression level of genes, reverse transcription, pre-amplification and quantitative PCR (qPCR) were performed. Sixty ng of extracted total RNA was reverse transcribed using OneTaq-PCR kit (New England Biolabs, Inc., Ipswich, MA, USA) and pre-amplified using TaqMan PreAmp mastermix (Applied Biosystems, Inc., Foster City, CA, USA; Thermo Fisher Scientific, Inc., Waltham, MA, USA). For the pre-amplification reaction, TaqMan Gene Expression Assays (Table 3) were pooled, followed by dilution to 0.2× using Tris-EDTA buffer solution, pH 8.0 (Sigma-Aldrich, St. Louis, MO, USA; Merck KGaA, Kenilworth, NJ, USA).

For the expression of microRNAs, TaqMan™ MicroRNA Reverse Transcription Kit (Applied Biosystems, Inc., Foster City, CA, USA) was used for the reverse transcription of the extracted total RNA following the provided protocol.

qPCR reactions were performed using TaqMan technology with FastStart Essential DNA Probe Master (Roche Diagnostics, Basel, Switzerland). Each qPCR reaction contained appropriately diluted cDNA, 2× FastStart Essential DNA Probe Master (Roche Diagnostics, Basel, Switzerland) and 20× TaqMan m(i)RNA assay, listed in Table 3. *IPO8*, *B2M* were used as reference genes for mRNAs expression and *RNU6B* and *hsa-miR-1274b* were used as reference genes for microRNAs expression. All qPCR reactions were conducted on a Rotor-Gene Q system (Qiagen GmbH, Hilden, Germany), and each sample was run in duplicate. The thermocycling conditions for gene expression were 2 min at 50 °C, 10 min at 95 °C and 40 cycles of 15 s at 95 °C and 1 min at 60 °C and for microRNA expression 10 min at 95 °C and 40 cycles of 15 s at 95 °C and 1 min at 60 °C.

To calculate the efficiency of qPCR reactions, pools of RNA samples of each group were created. RNA pools were reverse transcribed as described above, diluted in 5 steps, ranging from 4- to 1024-fold dilution and qPCR reactions were run in triplicate as described above.

### 4.5. Immunohistochemistry

We used commercially available antibodies against DCN, EPHA4, FN1, SPARC, SPON2, and SPP1 for immunohistochemical detection of aforementioned proteins. Detailed information about the used antibodies is presented in Table 4. Four μm thick sections were cut from paraffin blocks and deparaffinized, processed for antigen retrieval and stained in an automatic immunostainer (Ventana BenchMark; Roche Diagnostics, Basel, Switzerland) using UltraVIEW DAB Detection kit (Roche Diagnostics, Basel, Switzerland).

### 4.6. Statistics

First, we performed Fisher to test for heterogeneity of our samples. To calculate relative m(i)RNA expression, Cq values were corrected according to Latham et al. [56]. To obtain ∆Cq, the geometric mean of Cq values of the reference genes was deducted from Cq values of the genes (microRNA) of interest. Kruskal-Wallis test with post hoc Mann–Whitney U test was used to calculate statistically significant difference comparing ∆Cqs between different groups. Additionally, the Spearman’s Rank correlation coefficient (ρ) was used to calculate the statistical significance of association between the expression of analyzed genes and their regulatory microRNA (ΔCq values). All statistical analyses were performed with IBM SPSS Statistics 24.0 software (IBM Corp., Armonk, NY, USA) using cut-off value 0.0125.

## 5. Conclusions

Our study provides further evidence that ECM-related genes and proteins play a significant role in the early stages of the CRC development. Differences in the expression were observed in most of the analyzed ECM-related genes and proteins in AEM in comparison to AEC, reflecting inflammatory stromal reaction to traumatization and misplacement of dysplastic glands in the submucosa in the former, and desmoplastic stromal reaction to true invasion of dysplastic glands in the submucosa in the latter. Moreover, we experimentally confirmed a negative correlation between the expression of genes *DCN* and *SPP1* and their potential regulatory microRNAs in the development of CRC. Though the expression patterns of the analyzed genes and proteins are too complex to be used in diagnostic work, they might contribute to better understanding ECM changes in CRC development and might help to find new marker(s) of AEM and AEC in the future.

## Figures and Tables

**Table 1:** Gender and age of the patients included in the study.

Demographic Characteristics	Healthy Colon Mucosa	Adenoma	Adenoma with Epithelial Misplacement	Adenoma with Early Carcinoma	Advanced Carcinoma
male:female	10:11	10:0	10:3	6:4	7:4
Age (mean ± SD)	48–90 (75 ± 11)	50–83 (65 ± 11)	47–78 (58 ± 8)	56–72 (65 ± 5)	58–90 (76 ± 11)

**Table 2:** Results of database search for regulatory microRNAs of ECM-related genes.

microRNA	Gene	TarBase [13]	TargetScan [14]	miRTar [15]	miRNA & CRC [16]	Literature
*hsa-miR-200c*	*DCN*	-	-	-	+	[17]
*hsa-miR-200b*	*FN1*	+	-	-	+	[18,19]
*hsa-miR-200c*	*FN1*	+	-	-	+	[20,21]
*hsa-let-7a*	*EPHA4*	-	+	-	+	[22]
*hsa-miR-211*	*SPARC*	-	-	+	+	[23]
*hsa-miR-29a*	*SPARC*	+	+	+	+	-
*hsa-miR-29b*	*SPARC*	+	+	+	+	[24,25]
*hsa-miR-29c*	*SPARC*	+	+	+	+	[26]
*hsa-miR-493*	*SPON2*	-	-	-	-	[27]
*hsa-miR-146a* [12]	*SPP1*	-	-	+	+	-
*hsa-miR-127* [12]	*SPP1*	-	-	+	+	[28]
*hsa-miR-299* [12]	*SPP1*	-	-	+	+	[29]

**Table 3:** List of used TaqMan assays.

Gene or microRNA	Assay ID
*B2M*	Hs 99999907_m1
*IPO8*	Hs 00183533_m1
*DCN*	Hs 00266491_m1
*EPHA4*	Hs 00953178_m1
*FN1*	Hs 01549976_m1
*SPARC*	Hs 00234160_m1
*SPP1*	Hs 00959010_m1
*SPON2*	Hs 01557678_g1
*RNU6B*	001093
*hsa-miR-1274b*	002884
*hsa-miR-29a-5p*	002112
*hsa-miR-29b-5p*	00041
*hsa-miR-29c-5p*	000587
*hsa-miR-127-5p*	002229
*hsa-miR-146a-5p*	000468
*hsa-miR-200b*	002251
*hsa-miR-200c*	002300
*hsa-miR-211*	000514
*hsa-miR-299-5p*	000600
*hsa-miR-493*	001040
*hsa-let-7a*	000377

**Table 4:** Detailed information about the used antibodies for immunohistochemistry.

Antibody	Manufacturer	Catalogue Number	Type	Host Species	Dilution	Antigen Retrieval	Duration of Primary Antibody Incubation (T = 37 °C)
DCN	LifeSpan Biosciences	LS-B4312-50	Monoclonal	Mouse	1:1000	No pretreatment	12 min
EPHA4	SantaCruz Biotechnology	SC-365503	Monoclonal	Mouse	1:500	Cell conditioning solution 1 (Ventana BenchMark)	12 min
FN1	Cell Signaling	26836S	Monoclonal	Rabbit	1:300		28 min
SPARC	Invitrogen, Thermo Fisher Scientific, Inc.	33-5500	Monoclonal	Mouse	1:400		24 min
SPON2		PA5-59087	Polyclonal	Rabbit	1:20		32 min
SPP1		MA5-17180	Monoclonal	Mouse	1:300		24 min

## Supplementary Materials

The following are available online at https://www.mdpi.com/2072-6694/12/12/3715/s1, Figure S1: Expression of potential regulatory microRNAs that were not expressed in all groups. Expression is plotted as fold change against healthy colon mucosa. Legend: AD, adenoma; AEM, adenoma with epithelial misplacement; AEC, adenoma with early carcinoma; AC, advanced carcinoma.
