# Supplementary material for: Expression of Extracellular Matrix-Related Genes and Their Regulatory microRNAs in Problematic Colorectal Polyps
Source: Cancers (Basel). 2020 Dec 11;12(12):3715. doi: 10.3390/cancers12123715 (PMC7764749; doi:10.3390/cancers12123715)
Supplement: Supplementary file 1 [file cancers-12-03715-s001.pdf]

cancers

MDPI

Article

Expression of Extracellular Matrix-Related Genes and Their Regulatory microRNAs in Problematic Colorectal Polyps

Margareta Žlajpah, Emanuela Boštjančič, Bojan Tepeš and Nina Zidar

Supplementary Materials:

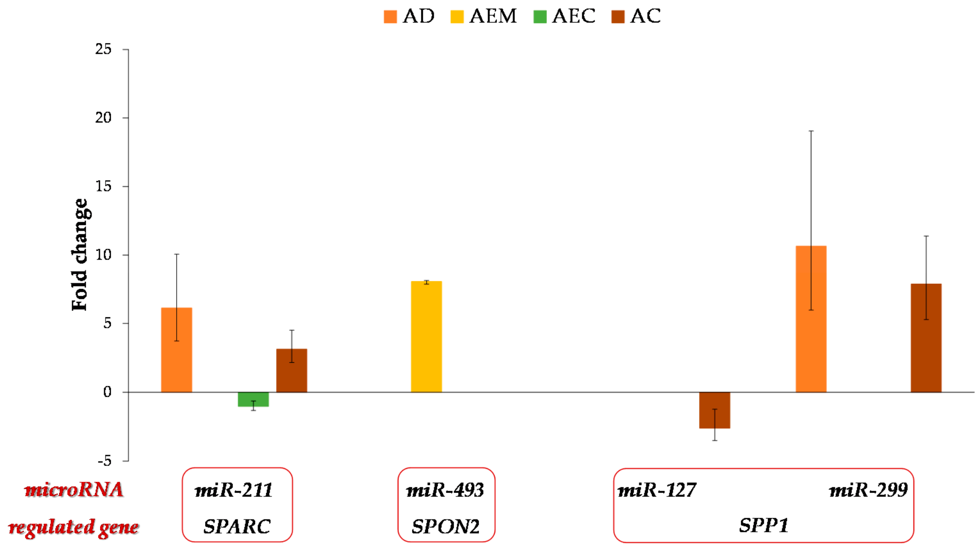

Figure S1. Expression of potential regulatory microRNAs that were not expressed in all groups. Expression is plotted as fold change against healthy colon mucosa. Legend: AD, adenoma; AEM, adenoma with epithelial misplacement; AEC, adenoma with early carcinoma; AC, advanced carcinoma.

© 2020 by the authors. Licensee MDPI, Basel, Switzerland. This article is an open access article distributed under the terms and conditions of the Creative Commons Attribution (CC BY) license (http://creativecommons.org/licenses/by/4.0/).
